# Radiomics in Determining Tumor-to-Normal Brain SUV Ratio Based on ^11^C-Methionine PET/CT in Glioblastoma

**DOI:** 10.17691/stm2023.15.1.01

**Published:** 2023-01-28

**Authors:** G.V. Danilov, D.B. Kalayeva, N.B. Vikhrova, T.A. Konakova, A.I. Zagorodnova, A.A. Popova, A.A. Postnov, S.V. Shugay, I.N. Pronin

**Affiliations:** Scientific Board Secretary, Head of the Laboratory of Biomedical Informatics and Artificial Intelligence; N.N. Burdenko National Medical Research Center for Neurosurgery, Ministry of Health of the Russian Federation, 16, 4^th^ Tverskaya-Yamskaya St., Moscow, 125047, Russia;; Medical Physicist; N.N. Burdenko National Medical Research Center for Neurosurgery, Ministry of Health of the Russian Federation, 16, 4^th^ Tverskaya-Yamskaya St., Moscow, 125047, Russia; PhD Student; National Research Nuclear University MEPhI, 31 Kashirskoe Shosse, Moscow, 115409, Russia;; Radiologist; N.N. Burdenko National Medical Research Center for Neurosurgery, Ministry of Health of the Russian Federation, 16, 4^th^ Tverskaya-Yamskaya St., Moscow, 125047, Russia;; PhD Student; N.N. Burdenko National Medical Research Center for Neurosurgery, Ministry of Health of the Russian Federation, 16, 4^th^ Tverskaya-Yamskaya St., Moscow, 125047, Russia;; Student; Pirogov Russian National Research Medical University, 1 Ostrovitianova St., Moscow, 117997, Russia;; Student; Pirogov Russian National Research Medical University, 1 Ostrovitianova St., Moscow, 117997, Russia;; Researcher; N.N. Burdenko National Medical Research Center for Neurosurgery, Ministry of Health of the Russian Federation, 16, 4^th^ Tverskaya-Yamskaya St., Moscow, 125047, Russia; Associate Professor; National Research Nuclear University MEPhI, 31 Kashirskoe Shosse, Moscow, 115409, Russia; Researcher; Lebedev Physical Institute of the Russian Academy of Sciences, 53 Leninskiy Prospect, Moscow, 119991, Russia; Head of the Project; Research Institute of Technical Physics and Automation, 46 Varshavskoye Shosse, Moscow, 115230, Russia; Professor, Academician of the Russian Academy of Sciences, Deputy Director for Research, Head of the Unit for X-ray and Radioisotopic Methods of Diagnosis; N.N. Burdenko National Medical Research Center for Neurosurgery, Ministry of Health of the Russian Federation, 16, 4^th^ Tverskaya-Yamskaya St., Moscow, 125047, Russia;; Pathologist; N.N. Burdenko National Medical Research Center for Neurosurgery, Ministry of Health of the Russian Federation, 16, 4^th^ Tverskaya-Yamskaya St., Moscow, 125047, Russia;

**Keywords:** glioblastoma, ^11^C-methionine PET/CT, TNR, radiomics, machine learning, artificial intelligence

## Abstract

Modern methodology of PET/CT quantitative analysis in patients with glioblastomas is not strictly standardized in clinic settings and does not exclude the influence of the human factor. Methods of radiomics may facilitate unification, and improve objectivity and efficiency of the medical image analysis.

**The aim of the study** is to evaluate the potential of radiomics in the analysis of PET/CT glioblastoma images identifying the relationship between the radiomic features and the ^11^С-methionine tumor-to-normal brain uptake ratio (TNR) determined by an expert in routine.

**Materials and Methods:**

PET/CT data (2018–2020) from 40 patients (average age was 55±12 years; 77.5% were males) with a histologically confirmed diagnosis of “glioblastoma” were included in the analysis. TNR was calculated as a ratio of the standardized uptake value of ^11^C-methionine measured in the tumor and intact tissue. Calculation of radiomic features for each PET was performed in the specified volumetric region of interest, capturing the tumor with the surrounding tissues. The relationship between TNR and the radiomic features was determined using the linear regression model. Predictors were included in the model following correlation analysis and LASSO regularization. The experiment with machine learning was repeated 300 times, splitting the training (70%) and test (30%) subsets randomly. The model quality metrics and predictor significance obtained in 300 tests were summarized.

**Results:**

Of 412 PET/CT radiomic parameters significantly correlated with TNR (p<0.05), the regularization procedure left no more than 30 in each model (the median number of predictors was 9 [7; 13]). The experiment has demonstrated a non-random linear correlation (the Spearman correlation coefficient was 0.58 [0.43; 0.74]) between TNR and separate radiomic features, primarily fractal dimensions, characterizing the geometrical properties of the image.

**Conclusion:**

Radiomics enabled an objective determination of PET/CT image texture features reflecting the biological activity of glioblastomas. Despite the existing limitations in the application, the first results provide a good perspective of these methods in neurooncology.

## Introduction

Glioblastoma is the most common primary malignant cerebral astrocytic neoplasm [1]. It is characterized by intratumor heterogeneity at the cytological, transcriptional, and genomic levels, which determines individuality of its molecular profile, the related prognosis, and the management [2].

A complex of neuroimaging modalities is used for glioblastomas including magnetic resonance imaging (MRI) and positron emission tomography (PET) combined with computed tomography (CT) [3]. PET/CT is an imaging technique with radiopharmaceuticals containing a radionuclide label (isotope). The uptake of this preparation in tissues enables the identification of tumor aggression, making PET/CT essential in neurooncology, especially for malignant neoplasm imaging.

Traditionally, the quantitative analysis of PET signal intensity is conducted by calculating a standardized uptake value (SUV) of a radiopharmaceutical in a specified tissue volume within the region of interest set by an expert. Comparing SUV values in the lesion and intact brain area, a relative tumor-to-normal brain uptake ratio (TNR), characterizing the malignity of the oncological process, is estimated. Considering the variability of SUV computation methods and the participation of an expert in the process, the PET/CT results assessment may be influenced by a subjective human factor. All of the above-said shows the importance of standardization and automation of an image-driven radiopharmaceutical uptake computation.

In recent years, searching for advanced informativity and objectivity in interpreting medical imaging, investigators have paid greater attention to radiomics, a new trend in quantitative image analysis. This evolving field of computer sciences is discovering the relations between the quantitative features of medical images and clinical information, including histological and genetic data, functional status, patient life expectancy, etc. [4]. The main purpose of these studies is to seek additional clinically relevant information in the image textures, which could essentially widen the capabilities of the current medical imaging. The radiomics toolbox enables the non-invasive studies of imaging correlates of the tumor’s biological properties. Using extracted image features for classification and regression with machine learning is also a prospect for partial or complete automation of labor-consuming imaging postprocessing and detection of sophisticated patterns unavailable to the naked eye that must generally improve personalized diagnosis and disease prognosis [5-7].

Based on digital image analysis, the statistics of voxel values distribution are estimated. These are also called features, parameters, and image biomarkers in radiomics research, and in the context of subsequent applications in machine learning, they may be called variables or predictors. In the present work, we call these primary sets of statistics the radiomic features.

In neurooncology, the studies evaluating the clinical significance of radiomic features from PET/CT images in patients with high-grade glial tumors are not numerous. We did not find any publications analyzing image correlates of the glioblastoma biological properties using the ^11^С-methionine PET/CT data with radiomics.

**The aim of the study** was to evaluate the potential of radiomics in the analysis of PET/CT glioblastoma images identifying the relationship between the radiomic features and the ^11^С-methionine TNR determined by an expert in routine.

## Materials and Methods

The preoperative PET/CT data in 40 patients (31 men, 77.5%, and 9 women, 22.5%) were obtained in our study. The average age was 55±12 years. All patients were treated at the N.N. Burdenko National Medical Research Center for Neurosurgery (Moscow, Russia).

The inclusion criteria in our study were as follows:

the diagnosis of “glioblastoma” verified by histologic and molecular methods;

supratentorial tumor location;

patient’s age of 18 years and older;

PET/CT brain scanning prior to neurosurgical treatment;

brain MRI prior to neurosurgical treatment;

no treatment for glioblastoma before PET/CT and MRI examinations;

informed consent signed by the patient;

PET performed in 2018–2020.

The study complies with ethical principles of the Declaration of Helsinki developed by the World Medical Association (2013).

In the preoperative period, all patients underwent brain MRI on the Signa HDxt tomograph (General Electric, USA) with magnetic field of 3.0 T and 8-channel head coil. The following pulse sequences (modes) were used:

T1 FSPGR BRAVO with isotropic voxel of 1×1×1 mm before and after intravenous contrast;

Т1 FSE in the axial projection with a slice thickness of 5 mm and 1-mm gap between the slices before contrast enhancement and T1 in the axial projection after contrast enhancement;

T2 FSE in the axial projection with a slice thickness of 5 mm and 1-mm gap between the slices;

T2 FLAR in the axial projection with a slice thickness of 5 mm and 1-mm gap between the slices;

DWI ASSET with a slice thickness of 5 mm and 1-mm gap between the slices with the apparent diffusion coefficient (ADC) mapping;

ASL perfusion (with CBF mapping).

^11^C-methionine PET/CT examination was performed on the Siemens Biograph 40 system (Siemens Healthineer, USA) using a standard protocol: 10-min scanning 10 min after intravenous injection of the preparation. Images were reconstructed using OSEM (ordered subset expectation maximization) algorithm with 5 iterations and 8 subsets and correction of the uptake with the low-dose CT scan.

To assess the metabolic activity of ^11^C-methionine in the tumor, mean values of SUV were calculated in 1.0 cm^3^ of the most active tumor part (SUVt) and in 1.0 cm^3^ of the normal cerebral tissue of the frontal lobe in the contralateral hemisphere (SUVn). The TNR was estimated as TNR=SUVt/SUVn.

To calculate the radiomic parameters, MRI was preliminarily co-registered with PET/CT using the PMOD v. 4.0 software (Switzerland). A single rectangular region of interest of the fixed size was installed on all the slices of the co-registered images in such a way that it covered the maximal tumor volume on any slice obtained by MRI and PET/CT. In this way, a full volume of tumor in 3D projection was encapsulated in a parallelepiped. After that, PET/CT data for each patient in the volume of the specified parallelepiped was exported to a file in the NIfTI format, which was used to calculate radiomic features.

### Calculation of radiomic features

The data were processed and analyzed using the R (v. 4.2.2) programming language in the RStudio Server IDE (RStudio version 2022.07.0+548) on the NVIDIA DGX A100 supercomputer (NVIDIA, USA). Radiomic features were calculated based on PET/CT with RIA library [8]. Calculations for each patient were performed for the complete image from the NIfTI file without supplementary masks. Image preprocessing included discretization of voxel values by 128 levels. The levels were formed in two ways: equally sized (“es_128”) and equal by the number of values entering the level, equally probable (“ep_128”). The choice of discretization levels was determined by a compromise between the computation speed and the expected value of the radiomic features.

The following groups of features were calculated [9]:

the first order statistics — the characteristics of signal intensity distribution without taking into account spatial features (mean, median, mode, etc.);

statistics calculated from the gray level co-occurrence matrix (GLCM) in the neighboring voxels in the given direction and at a given distance (contrast, homogeneity, difference, etc.);

statistics calculated by the gray level run length matrix (GLRLM) — gray level non-uniformity, run length non-uniformity;

statistics depending on the geometric texture features (volume, surface, surface-to-volume ratio, fractal dimensions, etc.).

### Statistical data analysis

The first step of the statistical analysis was to evaluate the linear relationship of each radiomic parameter with the TNR of ^11^С-methionine using the Pearson correlation coefficient. In the second step, linear regression models were built using glmnet library with radiomic features as predictors and TNR as a target (dependent) variable. To reduce the dimensionality of the feature space essentially, only those variables for which correlation with TNR was statistically significant (p<0.05) were included in the model. Further dimensionality reduction was done using LASSO regularization. The predictors were normalized before training: centered (subtraction of the predictor mean) and scaled (divided by predictor standard deviation). The target variable was transformed with decimal logarithm.

Machine learning was carried out in the series of tests according to the following algorithm. In each test, the training and test samples were formed randomly as 70 and 30% of the initial dataset, respectively. After the training, the true logarithmic values and the model’s predictions on the test sample were potentiated. Then, the prediction quality metrics that is mean absolute error, MAE, root mean squared error, RMSE, and predictor significance (absolute coefficients value) were calculated, and the list of these predictors was saved. The test was repeated 300 times. The distribution of prediction quality metrics was obtained with the frequency of certain predictors’ occurrence in the models.

The descriptive distribution statistics are presented in this paper as mean values ± standard deviation (M±σ) and also as median, 25^th^ and 75^th^ percentiles (Me [25%; 75%]). The correlation was assessed using Pearson and Spearman coefficients. The null hypothesis in statistical tests was rejected at the level of significance p<0.05.

## Results

**Figure 1.**
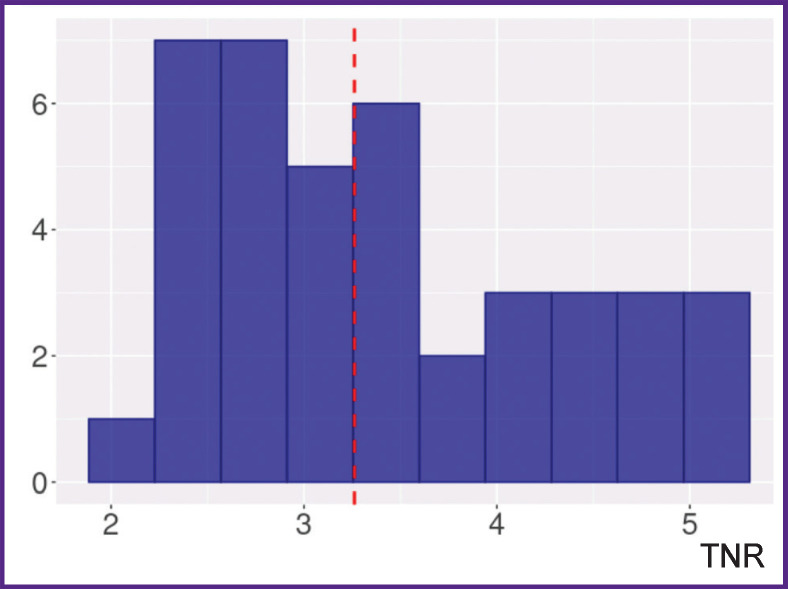
Histogram of ^11^С-methionine TNR distribution in the examined group of patients The red dotted line shows the median tumor-to-normal brain uptake ratio (TNR)

The distribution of TNR in the examined patients is presented in Figure 1. The median TNR was 3.26 [2.74; 4.17], and the minimum and maximum values amounted to 1.94 and 5.03.

**Figure 2.**
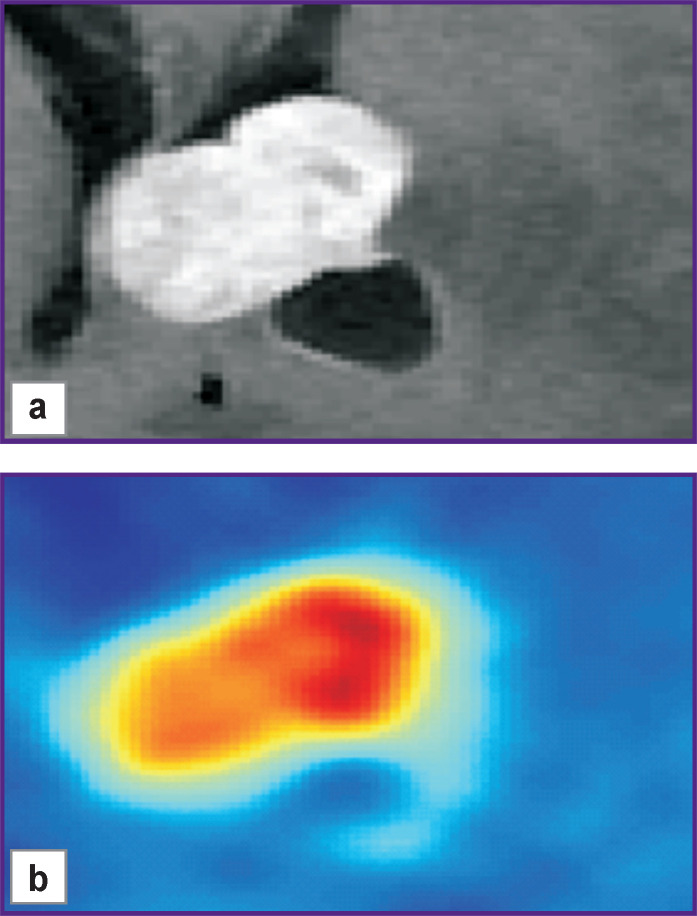
Example of one level in the region of interest set for PET radiomic features calculation in the patient with the diagnosis “glioblastoma localized in the lateral ventricles”: (a) MRI, T1 contrast; (b) ^11^С-methionine PET/CT

An example of the axial slices in the region of interest on the co-registered images from different modalities (PET/CT and MRI), which was used to calculate PET/CT radiomic features, is given in Figure 2.

**Figure 3.**
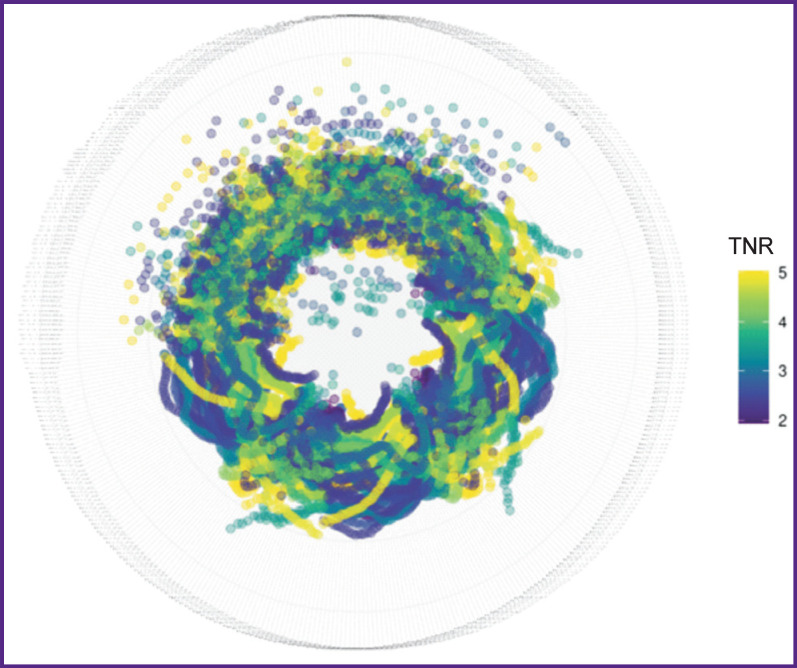
Radiomic features visualized in the polar coordinate system and scaled in the range from 0 (the circle center) to 1 (circumference of maximum radius) Color marks the value of the tumor-to-normal brain uptake ratio (TNR)

As a result of using radiomics over the initial and discretized PET images, 1362 quantitative parameters containing non-empty values have been obtained, of which 412 correlated with ^11^С-methionine TNR statistically significantly (median correlation coefficient was 0.44 [0.39; 0.54], median p-value was 0.0044 [0.0003; 0.0140]). The values of these features, scaled in the range from 0 to 1, are shown in Figure 3.

In each of the 300 machine learning tests, the training sample size was 28 cases, and that of the test sample was 12 cases. Below, we give prediction quality metrics for linear regression models generalized according to the results of 300 tests and calculated exclusively on the test samples.

The experiment showed the MAE median equal to 0.63 [0.51; 0.73], RMSE median — 0.87 [0.66; 1.09]. The median of the Spearman correlation coefficient between the true and predicted TNR in 300 tests was 0.58 [0.43; 0.74], and the median of the p-value was 0.05 [0.01; 0.16].

**Figure 4.**
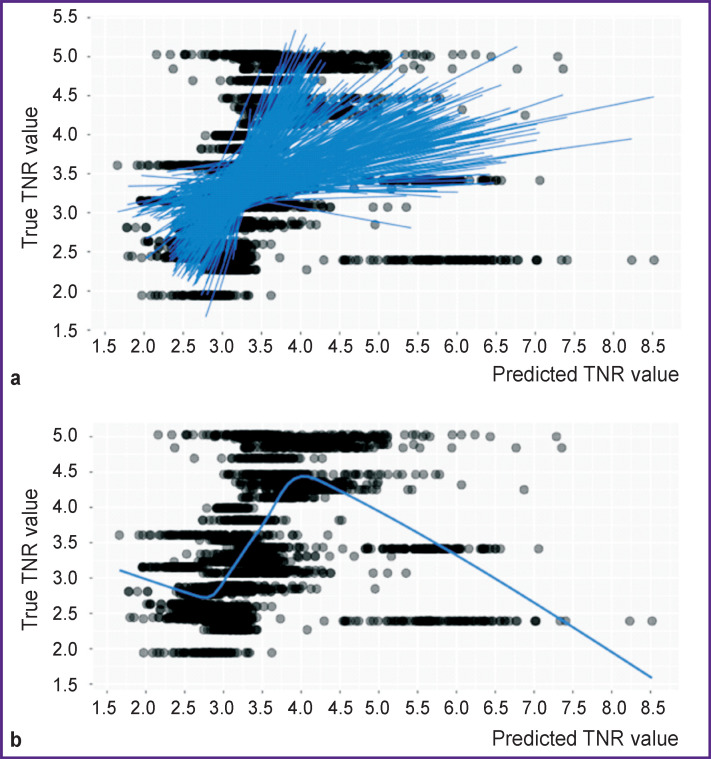
Compliance of true and predicted values of tumor-to-normal brain uptake ratio (TNR) in 300 tests: (a) blue color shows regression lines for all tests; (b) blue color shows the LOESS line for the entire dataset in 300 tests

In Figure 4 (a), the superimposed plots show the true and predicted TNR values fitting in 300 tests with appropriate regression lines. Figure 4 (b) illustrates a generalized tendency of the true and predicted TNR coincidence across all tests.

The median number of predictors utilized in each of the 300 models amounted to 9 [7; 13]; the minimum number of predictors was equal to 0, and the maximum to 30. Predictors most frequently (50 times and more) selected by the models are presented in the Table.

**Table:** Predictors selected 50+ times in the linear regression models with LASSO regularization (results of 300 tests; predictor names formed by the RIA library are preserved)

No.	Coded predictor identification	Number of models	Model share (%)	Mean significance
1	fractal_bc_d_22__es_128	157	52.3	0.0274
2	fractal_bc_d_121__es_128	122	40.7	0.0172
3	fractal_bc_d_40__es_128	117	39.0	0.0160
4	fractal_bc_d_113__es_128	86	28.7	0.0165
5	Cluster_p_s_nd__ep_b128_d1_mean	82	27.3	0.0117
6	Inv_Gauss_2p_s__ep_b128_d1_mean	81	27.0	0.0173
7	fractal_bc_d_36__es_128	77	25.7	0.0149
8	fractal_bc_d_123__es_128	66	22.0	0.0127
9	fractal_c_d_126__es_128	62	20.7	0.0180
10	Max_AD_md__orig	60	20.0	0.0133
11	Homogeneity2_e_nd__es_b128_d1_mean	57	19.0	0.0098
12	fractal_bc_d_24__es_128	54	18.0	0.0221
13	Cluster_d_s__es_b128_d1_mean	51	17.0	0.0108
14	Inv_Gauss_2f_s__ep_b128_d1_mean	50	16.7	0.0126

The main elements of predictor names from the Table are explained below.

The common elements in the names of all variables presented in the Table are as follows:

“__es” — a type of discretization with equal size of bins for gray levels (equally sized);

“__ep” — a type of discretization with equal probability of signal intensity values entering every gray level (equally probable);

“__orig” — calculation using original non-discretized image;

“_128” — total number of gray levels obtained by image discretization.

Fractal dimensionality names (geometric characteristics of the images) include the following designations:

“fractal” — a common designation of a fractal dimension;

“_bc” — Minkowski dimension (calculated by box-counting algorithm);

“_c_d” — a correlation dimension;

number (from 1 to 128) — the gray level for which fractal dimension is calculated.

The following components are used in the names of statistics calculated from the gray level co-occurrence matrix:

“Cluster_p_s_nd” — cluster prominence non-diagonal;

“Cluster_d_s” — cluster difference;

“Inv_Gauss_2p_s” — inverse Gaussian 2 polar;

“Inv_Gauss_2f_s” — inverse Gaussian 2 focus;

“Homogeneity2_e_nd” — homogeneity^2^ non-diagonal [9].

The only statistic of the first order from the Table has “Max_AD_md” designation — maximum absolute deviation from the median.

## Discussion

Radiomics in neurooncology is an actively evolving scientific area. The typical application of radiomics methods in glioblastoma imaging is a differential diagnosis (with other tumors, pseudoprogression), survival prognosis in general life expectancy, identification of molecular biomarkers (for example, IDH1, MGMT) [10]. However, the number of studies in which radiomics is used to investigate PET/CT images of brain glioblastoma is small.

In a study by Cao et al. [11], the authors solved the task of differentiating glioblastoma and solitary metastases using radiomic features of images obtained by MRI and ^18^F-fluorodeoxyglucose PET and demonstrated high model quality metrics. Similar high indicators of model performance were demonstrated by Zhang et al. [12], using the data obtained from MRI with contrast-enhancement and in the diffusion mode in addition to the PET data. Li et al. [13] showed the possibility of combining clinical parameters and radiomic features from dynamic О-(2-[^18^F]-fluoroethyl)-l-tyrosine PET in predicting the survival of patients with glioblastomas, IDH wild type, with moderate model quality. Barry et al. [14] studied the reproducibility of radiomic features in repeated О-(2-[^18^F]-fluoroethyl)-l-tyrosine PET. Carles et al. [15] have shown that radiomics gives essential information for prognosis in patients with recurrent glioblastoma. Some experience in the differential diagnosis of the true tumor progression and pseudoprogression has been obtained by Lohmann et al. [16]. An example of differential diagnosis of glioblastomas and lymphomas based on PET data with radiomics has been presented by Kong et al. [17]. Qian et al. [18] have demonstrated the possibility of determining the MGMT status in patients with glioblastomas by the PET data using radiomics.

A common restriction of current research in radiomics of PET/CT glioblastoma images is a sample size that rarely exceeds a hundred observations. Besides, the machine learning quality in these works does not always reach a high level. In studies with this relatively low-volume data, heterogeneity and sample imbalance may occur, leading to the failure to reach maximum model performance and built decisions keeping the adequate performance on new independent series of observations [10].

Considering the scientific literature data, we believe this article presents the first study that applies radiomics to analyze imaging correlates of biological glioblastoma features using ^11^С-methionine PET/CT. Although the size of our image sample is limited, the first results ground further analysis as more data become available and supplementary clinical information is included. Thus, on the lower half of the “ring” (Figure 3), we can see apparent visual patterns of TNR distribution, which are related to fractal dimensions. The existence of these patterns suggests their separability. In the case of pattern stability and accumulation of a significant number of observations, the entire potential of machine learning may be involved in their separation.

Figure 4 (b) shows a steady linear trend for all 300 tests — a high correlation of the true and predicted values in the mid-range of TNR distribution coinciding approximately with the interquartile range. The stability of this trend in our study is another reason to reproduce experiments on a large-volume sample.

We have established that geometric parameters of ^11^С-methionine PET/CT image, namely, fractal dimensions, were the most significant predictors of ^11^С-methionine TNR. Besides, these variables appeared in the models in each 2^nd^–3^rd^ test for several gray levels.

An essential aspect of this work is the absence of substantial image preprocessing and maximal usage of a vast region of interest, including the visible tumor volume and signals from the surrounding visually intact brain structures. In some cases, an air space beyond the skull appeared in the region of interest. Our study showed that this approach to the region of interest selection does not interfere with the indirect TNR assessment by the PET/CT radiomics with the linear regression model. This is probably facilitated by the inclusion of intact brain areas, which are used in the standardized methodology of TNR estimation.

### Limitations of the present study

The main limitation of our study is a small sample size and an incommensurably large number of radiomic parameters. The sample was not enriched with artificial, synthetic techniques. A small sample size is likely to explain a great variability of the results in 300 tests, with the division into training and test subsets. Multiple repetitions of machine learning with the selection of training and test samples are an important methodological aspect of our work since it prevents accidental overrating or underrating of the expected modeling quality metrics at such a small data volume.

The region of interest selection for radiomics computation was performed by an expert, and it varied depending on the tumor localization and size. It resulted in the regions of interest being unequal in size and heterogeneous in their content.

Limitations of the study are also connected with the choice of only one discretization variant and the lack of other calculation parameters variation. Part of the radiomic features was excluded from the analysis due to missing values. The splitting into the training and test samples at a small volume of investigations was likely to influence the quality of machine learning and the results of distinct tests.

All the above restrictions are typical for studies in radiomics. Further efforts will be directed toward overcoming these limitations by obtaining, for example, a greater volume of data.

## Conclusion

Radiomics enables the objective determination of PET/CT texture features reflecting the biological activity of glioblastoma and can potentially augment the efficiency of neuroimaging. Despite the existing limitations in radiomics application, the first results demonstrate promising prospects for their development. The existence of regularities revealed on the small PET/CT samples must be verified on large datasets.
